# Structural optimization and prospect of constructing hemoglobin oxygen carriers based on hemoglobin

**DOI:** 10.1016/j.heliyon.2023.e19430

**Published:** 2023-08-29

**Authors:** Yuexiang Ma, Qi Zhang, Zheng Dai, Jing Li, Wenxiu Li, Chuanqing Fu, Qianmei Wang, Wen Yin

**Affiliations:** a Department of Emergency, Xijing Hospital, Air Force Medical University, Xi'an, 710032, Shaanxi Province, China; b Shaanxi Provincial Regenerative Medicine and Surgical Engineering Research Center, First Affiliated Hospital of Xi'an Jiaotong University, Xi'an, 710061, Shaanxi Province, China

**Keywords:** Hemoglobin-based oxygen carriers, Chemical modification, Red blood cells, Organs storage

## Abstract

The current global shortage of organ resources, the imbalance in donor-recipient demand and the increasing number of high-risk donors make organ preservation a necessity to consider appropriate storage options. The current method of use often has risks such as blood group mismatch, short shelf life, and susceptibility. HBOCs have positive effects such as anti-apoptotic, anti-inflammatory, antioxidant and anti-proliferative, which have significant advantages in organ storage. Therefore, it is the common pursuit of researchers to design and synthesize HBOCs with safety, ideal oxygen-carrying capacity, easy storage, etc. that are widely applicable and optimal for different organs. There has been a recent advancement in understanding HBOCs mechanisms, which is discussed in this review.

## Graphical abstract

**Figure:**
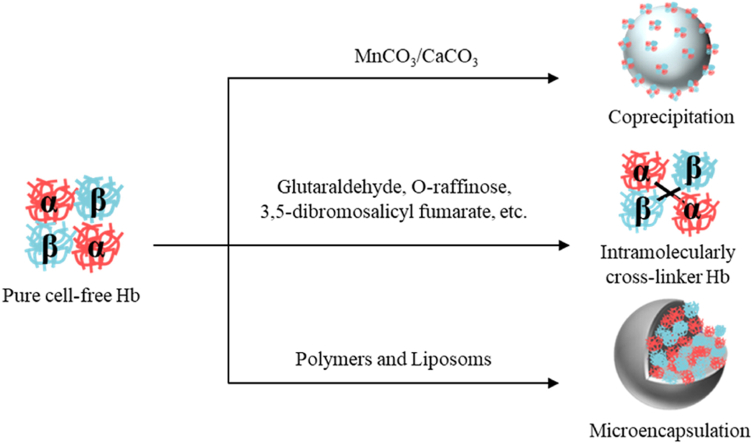

## Abbreviations

BHb: Bovine hemoglobin
ECD: Expanded criteria donor
DHSG: 1,5-*O*-dihexadecyl-*N*-succinyl-*l*-glutamate
DPPC: 1,2-dipalmitoyl-*sn*-glycero-3-phosphatidylcholine
EDTA: Ethylene diamine tetraacetic acid
Hb: Hemoglobin
HBOCs: Hemoglobin-based oxygen carriers
Hb-V: Hemoglobin vesicles
HHb: Human hemoglobin
HSA: Human Serum Albumin
IRI: Ischemia reperfusion injury
GA: Glutaraldehyde
LBL: Layer by layer
MQ: Milli-Q
nCV: Normalized cell viability
PDA: Polydopamine
PEG: Polyethylene glycol
PEG-DSPE: 1,2-distearoyl-*sn*-glycero-3-phosphatidylethanolamine-*N*-poly (ethylene glycol)
RBC: Red blood cells
SOT: Solid organ transplantation

## 1 Information

The increased success of solid organ transplantation (SOT), the treatment of choice for end-stage organ disease, has been hampered by ischemia-reperfusion injury (IRI) [1]. According to the data from the OPTN/SRTR 2021 Annual Data Report about Deceased Organ Donation, there were 13 862 deceased donors, a 10.1% increase from 12 588 in 2020, and an increase from 11 870 in 2019; this number has been increasing since 2010 [2]. IRI unavoidably occurs during organ resection and transplantation, may compromise the short-term and long-term after transplantation, and remains a critical organ transplantation challenge. With the increase in expanded criteria donor (ECD), the selection of better preservation methods to improve the preservation time of isolated organs, tissue oxygenation, etc., and to further reduce organ IRI remains an urgent issue [3].

The methods commonly used today to preserve organs are: under room temperature conditions using crystalloid and RBCs or under cryogenic conditions at 4 °C using clinically standard preservation solutions [4,5]. However, the above methods suffer from storage fluid cross-matching, scarcity, and infection-induced oxidative metabolism of the organ, which in turn exacerbates oxidative stress damage to the organ [6]. In addition, the limitation of the preservation fluid makes it necessary to use the organs in a short period of time, otherwise thousands of organs will be abandoned. Therefore, we have a critical need for better organ isolation preservation fluids to provide near physiological conditions for the duration of organ preservation, thereby improving the quality of transplanted organs to address the global crisis of organ shortage due to organ storage.

HBOCs (hemoglobin-based oxygen carriers) have expanded from their initial use as a blood substitute to ischemia and hypoxia therapy as a near physiologically conditioned oxygen carrier. Among the many properties that make hemoglobin (Hb) the ideal oxygen carrier within red blood cells are its oxygen affinity, long-term stability, stability of tetramers, and cooperativity [7,8]. During the past few decades, diverse nanoscale carriers have been developed for physical encapsulation or chemical conjugation of Hb, which were known as HBOCs [9]. To obtain desirable HBOCs for organ preservation, HBOCs of different particle sizes were synthesized in different buffers by chemical modification means, such as co-precipitation, desolvation, cross-linking, microencapsulation, selected from the above-mentioned methods (Table 1).

**Table 1:** Different HBOCs sources, solvents and particle size.

Polyhemoglobin	Source	various of buffers	Mean diameter	Ref.
Hb-MPs	BHb	EDTA solution (0.2 M, pH 7.4, 20 mL)	3.30 ± 0.80 μm	[10]
ZnPc-loaded HbMs	BHb	Phosphate buffer (10 mM, pH = 7.4)	66.01 ± 0.95 nm	[11]
PLGA^Hb^/M-NCs	BHb	Tris(hydroXymethyl) aminomethane (TRIS)	～95 nm	[[12], [13]]
PDA-Hb	BHb	Tris-HCl buffer (10 mM, pH 8.5)	3.32 ± 0.49 μm	[14,15]
Hb@lipo	HHb	pH 7.4 PBS	～120 nm	[16]
BP QDs-Hb-encapsulated biocompatible	HHb	Deionized water	NA	[17]
GelMA hydrogel				
Hb-PDA	BHb	Tris-HCl buffer (10 mM, pH 8.5)	6–8 nm	[18]
Hb-conjugated biotins	HHb	Phosphate buffer (50 mM, pH 6.5)	NA	[19]
SA@Hb@CQDs	HHb	Deionized water	NA	[20]
ZIF-8P-Hb	BHb	Deionized water	106.0 ± 9.7 nm	[21]
SFHbNP	HHb	Phosphate buffer (10 mM, pH 7.4)	～90 nm	[22]
HbAvHb	HHb	Phosphate buffer (50 mM, pH 6.5)	NA	[23]
SPolyHb	Guinea pig hemoglobin	Phosphate buffer (50 mM, pH 7.4)	＞500 kDa	[24]
Hb@lipo	HHb	phosphate-buffered (pH 7.4)	～120 nm	[25]
HEP	BHb	phosphate-buffered (pH 7.4)	～200 nm	[26]
Hb-PEG	HHb	Phosphate buffered (10 mM Na_2_HPO_4_, pH 7.4)	10.6 ± 3.6 nm	[27]
PolyhHbs	BHb	Phosphate buffered (10 mM, pH 7.4)	～80 nm	[28]
Hb-V	HHb	Phosphate buffered (10 mM, pH 7.4)	250–280 nm	[29]
Fe_3_O_4_-PEI-PA-Yb^3+^	BHb	Tris-HCl (pH 6)	～105 nm	[30]

In this review, we summarize the use of purified hemoglobin that was structurally modified for organ storage. Most examples presented here have been published within the past 5 years, and greater emphasis has been given to recent examples that are illustrative principles.

## 2 Materials of HBOCs-Hb

The function of Hb includes maintaining the pH of the blood, carrying oxygen to peripheral tissues, as well as transporting carbon dioxide. Structurally, hemoglobin chains retain the classic globin fold, which is shared by several proteins. Each hemoglobin chain harbors a heme group in a hydrophobic pocket. A ferrous ion (Fe^2+^) of the heme group associated with each hemoglobin chain acts as a cofactor for this tetrameric protein. An Hb molecule is also categorized as a supramolecular assembly that consists of two α and two β subunits (α2β2). They are assembled using a combination of non-covalent interactions as hydrogen bonds, hydrophobic forces, van der Waals forces, and electrostatic effects. Its tetrameric structure(α2β2) is fundamentally stable under physiological conditions, but dissociates reversibly into dimers, thereby exchanging dimers intermolecularly(αβ) [31]. Specifically, the amino acids that comprise each of the three coding sequences of Hb are responsible for the following functions: i) heme contacts essential for oxygenation, ii) contacts α1 and β1 are essential for a cooperative dimer, iii) α1-β1 contacts essential for cooperative tetramer, iv) the Bohr effect that modulates oxygen loading and unloading from the lung to tissues, and v) oxygen affinity regulation requires 2,3-diphosphoglycerate binding [32,33].

**Fig. 1:**
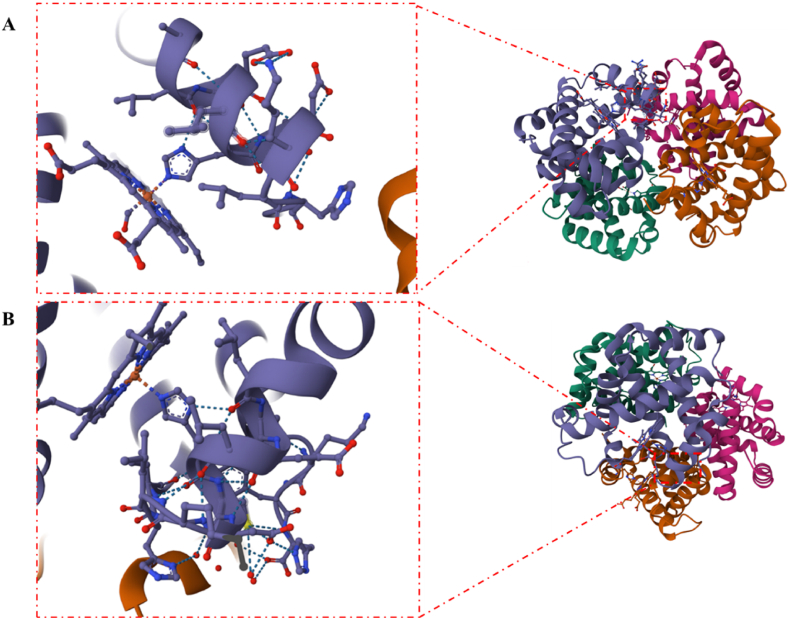
Comparison of the spatial structure of BHb and HHb. A. BHb and β-Cys_93; B. HHb and β-Cys_93 (PDB: 6IHX and 1A3N).

Due to its genetic similarity to human hemoglobin (HHb) at 90% of amino acid sequence and its better oxygen-carrying capacity, bovine hemoglobin (BHb) is commonly used in hemoglobin synthesis [34,35] (Fig. 1). Both BHb and HHb consists of two α-chains with 141 amino acid residues each and two β-chains with 146 amino acid residues each that form a tetrameric protein molecule [36]. In fact, Hes_63, His_92, and Phe_42 in beads proteins act as markers to maintain the position and orientation of the heme group, while Cys_93 in this protein uses NO as a site-modifying enzyme in Hb [37]. Although mammalian Hb contains six cysteines, all of which are capable of binding NO, β-Cys_93 has two unique properties (one in each α-globin and two in each β-globin): it is not only the most active Cys residue in Hb, but also its activity is related to the binding and release of oxygen at the heme site (“thermodynamic link”) [38,39].

## 3 Chemical modification

### 3.1 Coprecipitation

**Fig. 2:**
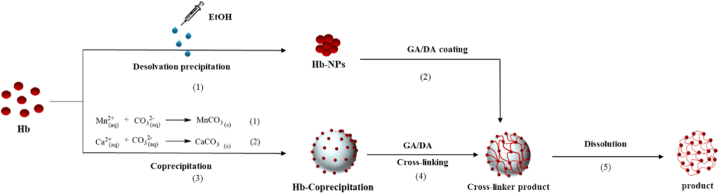
Synthesis of HBOCs based on co-precipitation method.

Since the affinity of neutral salt to water molecules is greater than that of Hb, it causes the hydration layer around protein molecules to weaken or even disappear. As the ionic strength changes with the addition of Hb to the neutral salt, the charge on the Hb surface is heavily neutralized, leading to a decrease in solubility and allowing co-precipitation by aggregation between molecules [38](Fig. 2). When a precipitant is added, homogeneous precipitation can be obtained. By adopting this method, precipitated particles with uniform chemical composition, small particle size, and uniform steps can be obtained. Carbonate ions (CO_3_^2−^) and nanoparticles are used as precipitants for co-precipitation. **a. MnCO**_**3**_: A combination of MnSO_4_ and NH_4_HCO_3_ was used to synthesize MnCO_3_ particles from Chunmei yu for its excellent absorption capacity [40]. As a result of their high oxygen affinity, PDA-Hb microcapsules can bind and reversibly release oxygen. Metal carbonates of MnCO_3_ are formed when MnCl_2_ reacts with Na_2_CO_3_, encapsulating Hb in M. Emily [41]. **CaCO**_**3**_: Using a co-precipitation process between Hb and CaCO_3_, followed by covalent sphere assembly between Hb and GA, Li and co-workers have recently developed highly loaded Hb spheres [14,15]. They used CaCO_3_ particles as templates for fabricating Hb spheres that contain a high loading content in order to exploit their special properties, such as porous channel-like structures and high surface areas. CaCl_2_ and Na_2_CO_3_ were co-precipitated to produce CaCO_3_ particles. For every CaCO_3_ particle, there were 1.36 g/cm^3^ of Hb.

**b. Desolvation precipitation**: Desolvation results in the effective supersaturation of a protein solution by replacing water with an antisolvent, which is insoluble in water [42]. Precipitates are commonly dissolved with polar solvents. By applying classical nucleation theory, precipitates are formed as protein-protein interactions take precedence over protein-solvent interactions as the antisolvent is added [43]. Chen and colleagues used a magnetic stirring apparatus to dissolve Hb with different concentrations of ultrapure water (MQ), using EtOH as an excipient for protein dissolution [44]. EtOH was then added dropwise to Hb solutions under continuous stirring at different volumes based on the water phase. In order to remove the turbid suspensions, a benchtop centrifuge was used and MQ washes were used to spin the suspensions down. However, sonication and resuspension of Hb-NPs were both successful. The method is simple to operate, does not introduce toxic solvents, has uniform particle size, high encapsulation efficiency, and good re-dispersibility in water, and is a relatively common method. The synthesis of HBOCs by coprecipitation usually controls the amount of Hb in the spheres by regulating the initial concentration of hemoglobin, which in turn improves the oxygen-carrying capacity. Nevertheless, the Hb loading efficiency in the spheres can also be adversely affected by high concentrations. In addition, the stability of HBOCs obtained by this method is relatively poor. Therefore, on this basis, other chemical modification methods were introduced for structural modifications.

### 3.2 Cross-linking

**Fig. 3:**
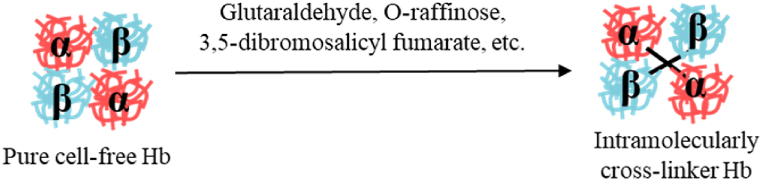
Synthesis of HBOCs based on cross-linking method.

**Fig. 4:**
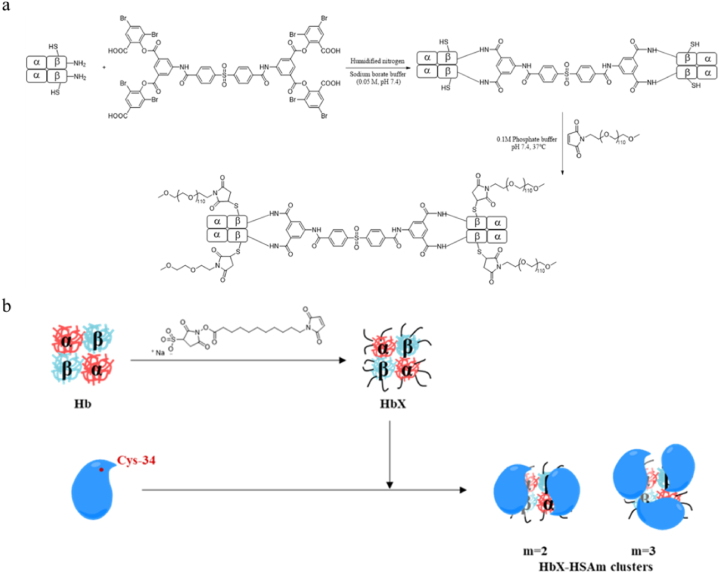
Hemoglobin oxygen carriers was synthesized by cross-linking method. a) Synthesis of hemoglobin oxygen carrier BT-HB-PEG5K4 based on the cross-linking method; b) Schematic illustrations of the synthetic route of the HbX-HSA_m_ cluster.

Cross-linking selects functionalities of the components to form polymers of a certain size by cross-linking with exposed amino acid residues –SH or –NH_2_ of Hb [45](Fig. 3). By increasing the particle size of Hb, cross-linking improves a certain oxygen-carrying capacity, increases the half-life of HBOC, and reduces the degradation of metabolic enzymes. However, to a certain extent, it causes side effects such as vasoconstriction. HBOCs are commonly used as a polymer of glutaraldehyde, polydopamine and glucan oxide and so on [[33], [34], [35], [36], [37], [38], [39], [40], [41], [42], [43], [44], [45], [46]]. The purified hemoglobin was dissolved in the anti-solvent ice ethanol, and oxidized dextran (2 wt%) was added for cross-linking reaction when the solution became turbidity. The reaction was completed by adding NaBH_3_CN to quench the reaction. Subsequently, the template is usually removed with EDTA or Na_2_EDTA after the cross-linked Hb is completed. It has been noted that hemoglobin's nitrite reductase activity is a potential source of biologically active NO during hypoxia and ischemia. Activating variable structure-controlled nitrite reductase with hemoglobin's heme fraction allows nitrite in the blood to be converted to NO [47,48]. Protein modifications that alter the binding affinity of the ligand to heme or its redox potential are considered to contribute to the enhancement of nitrite reductase activity. Ronald Kluger produced the PEGylated bis-tetramers with cross-linking that makes hemoglobin (BT-HB-PEG5K4)(Fig. 4a). In order to develop functional oxygen therapeutics that are effective, these polymers must have high oxygen affinity and nitrite reductase activity [49]. Their research has led to the production of PEGylated bis-tetramers of hemoglobin (BT-Hb-PEG5K4), enhancement of oxygen affinity, and improvement of nitrite reductase activity, all of which are critical for the development of functional oxygen therapies. Daiki Tomita used α-succinimidyl-ε-maleimide crosslinker constructs to covalently wrap Hb with HSAs to generate new core-shell protein clusters [50](Fig. 4b). O_2_-carrying HbX-HSAm clusters with negative surface net charges, high O_2_ affinity, and lower P_50_ value, does not cause vasoconstriction and NADH-dependent reductase unit shells can provide O_2_ therapeutic reagent in various clinical situations.

## 4 Microencapsulation

In order to further improve the oxygen capacity of the Hb vector and increase the function of the carrier, the researchers used the polymer and liposomes to encapsulate the hemoglobin, which formed the scale of the micro sodium level and made up a more stable and multivariate oxygen delivery system. These methods not only increase the quality of the hemoglobin package, but also improve the compatibility of the objects, and adapt the more complex biological environment to the prospect of a broader application of the hemoglobin vector.

### 4.1 Polymer

**Fig. 5:**
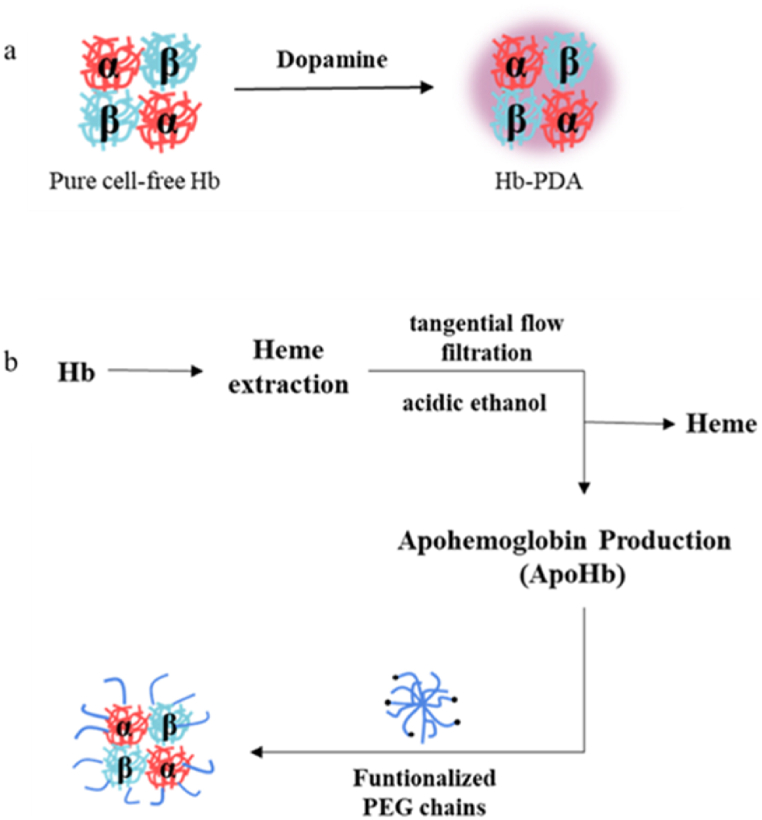
Synthesis of hemoglobin oxygen carrier in polymeric form. a) Synthetic pathway of Hb-PDA; b) Synthetic pathway of PEG-apoHb.

Polymersomes are of interest as nanocarriers due to their physical and chemical robustness, which arises from the macromolecular nature of their block copolymer components. The polymer of the polymerized hemoglobin: dextran, dopamine, PEG, *etc*. The polydopamine (PDA) coating material is highly adherent to all surface types of substrates and is simple and easy to apply. The PDA not only has little effect on the survival and proliferation of many kinds of mammal cells but also as an antioxidant agent, removing free radicals from distinct hydroquinone moiety. Platelet adhesion and fibrinogen conformation transition could be effectively inhibited by surfaces modified with PDA [[51], [52], [53]]. By encapsulating Hb in one step using a simple PDA coating, Wang and colleagues focused on the coating's desirable properties [54](Fig. 5a). An incubation mixture containing dopamine hydrochloride and Hb was incubated at room temperature for 3.5 h with slight stirring using a Tris-HCl buffer. The total volume of the reaction system was 2 mL, and it was dialyzed in PBS solution to remove excess dopamine hydrochloride. In addition, DA was oxidatively polymerized in TRIS 1 for coating Hb with PDA by Jansman [55]. Briefly, 8 mg/mL solution of Hb and 1.6 mg/mL solution of DA were mixed at 1:1 vol ratio in TRIS 1 and rotators for 3 h were used. The resulting Hb^PDA^ was washed in TRIS 2 using a bench-top centrifuge and amicon centrifugal filters. By conjugating polyethylene glycol (PEG) to the protein surface, protein therapeutics can increase their circulatory half-life in vivo. PEG is used as a standard for nanoparticle and protein coupling surfaces due to its excellent biocompatibility and increased hydration radius after coupling. Furthermore, PEG chains possess bristle-like structures on their molecular surfaces that prevent T-cell recognition and reduce clearance of PEG-coupled therapeutic molecules [56,57]. As a result of PEGylated apoHb (PEG-apoHb) coupling of PEG to apoproteins via thiol-maleimide, Ivans S and colleagues improved lipoprotein stability, circulating half-lives, and the prevention of PEG-apoHb extravasation [58](Fig. 5b).

### 4.2 Liposome

**Fig. 6:**
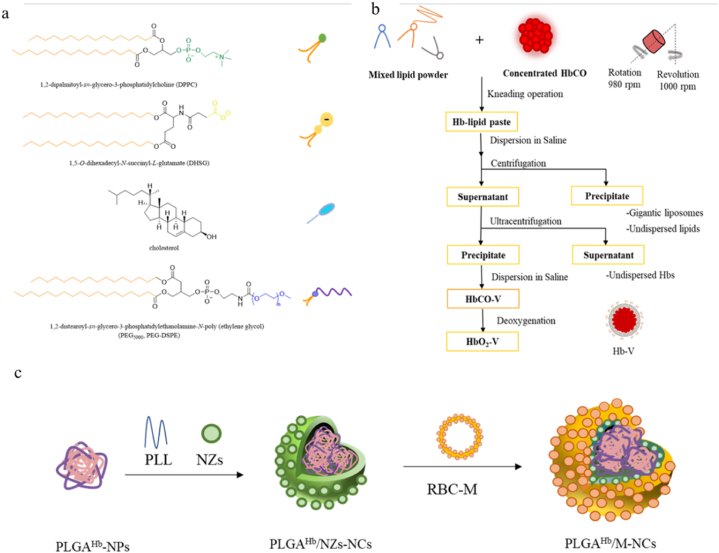
Synthesis of hemoglobin oxygen carrier in microcapsule form. a) Chemical structure of DPPC, DHSG cholesterol, and PEG-DSPE; b) Synthetic pathway of HbO_2_-V; c) Synthetic pathway of PLGA^Hb^/M-NCs.

Hb encapsulated in lipid membranes without antigen, and thereby reducing renal excretion, would not only increase circulation time, but eliminate the need for matching blood groups [22,59,60]. Despite PEG being the standard for delivering stealth properties to intravenously administered carriers, the production of anti-PEG antibodies is increasingly recognized as a concern. By creating PEG antibodies, the liver and spleen recognize and eliminate PEGylated drugs and NPs by creating mononuclear phagocytes (MPS). In order to avoid this drawback, alternative PEGylation techniques are highly sought after [[61], [62], [63]]. As the most common substitute for biological membranes, amphiphilic molecular phospholipid vesicles or liposomes self-assemble in water to form bilayers (Fig. 6a) [64]. The interaction with plasma proteins poses difficulties in controlling particle size and inhibiting aggregation [65]. Using the extrusion method, particles of phospholipids are dispersed in an aqueous phase and then extruded through filters with different pore sizes [66]. Viscosity increases further when lipids are added to hemoglobin vesicles (Hb-V) to enhance oxygen carrying capacity. Extrusion methods clog filters due to the mixture's high viscosity. The mixture of lipids was therefore limited [67]. It has been proposed that freeze-dried liposomes can be mixed to resolve this difficulty. However, Hb remains limited in how much lipid can be mixed with it. Tomoko Kure and Hiromi Sakai developed “dual (asymmetric) centrifugation (DAC or DC)" after carefully understanding the background and difficulties presented above [68]. Using planetary motion, raw materials are sealed inside a cylindrical container (vessel) that rotates around a central axis and a second axis simultaneously. To prepare the mixed lipids, specific molar ratios of DPPC, DHSG, and PEG-DSPE were dissolved in 2-methyl-2-propanol by stirring in a 500 mL flask at 60 °C(Fig. 6a and b). Then, the lipid mixture solution was freeze-dried for 1 day to obtain a powdered lipid mixture. Liposomal preparation can be performed in fewer steps with this mixer without contamination, rapidly and aseptically. In addition, Michelle Maria Theresia Jansman and his colleagues chose human blood cells as phospholipid membranes to wrap the Hb(Fig. 6c) [59]. Nanocarriers with membrane coatings were shown to be novel oxygen carriers that combine antioxidant and stealth properties.

## 5 Biocompatibility and hemocompatibility

^1^Good biocompatibility is a central aspect when developing HBOCs to preserve the normal physiological functions of the body [44]. According to the literature, HUVEC, RAW 264.7, HEK 293T, HepG 2 cell lines were usually selected for HBOCs for biocompatibility determination by CCK-8 and other methods. HbPDA was not significantly cytotoxic after 24 h incubation in different concentration ranges [18]. The biocompatibility of the **Hb/PDA-NPs** was evaluated by assessing the in vitro cell viability of HUVEC and RAW 264.7 cells [44]. It was shown to inhibit cells by approximately 15% at only higher concentrations of 30 000 NPs/μL. The PDA-Hb microcapsules designed by Yu C did not produce significant cytotoxic activity on HEK 293T cells compared to the control [40]. The IL/PDA nanocapsules studied by Tan et al. still showed 80% cell viability at 200 μg/mL. Therefore, it was shown to have no significant cellular activity [53][69]. RAW264.7 cells in PLGA^Hb^/M − NCs showed a significant decrease in cellular activity with increasing concentrations of NCs, whereas this did not occur in HUVEC. Leticia Hosta-Rigau suggested that the decrease in RAW264.7 cellular activity was the result of phagocytosis of NCs by cells, independent of cell membrane concentration [55]. In addition, the study by Leticia Hosta-Rigau's group found no significant differences based on different coating methods to cover Hb.

Hemocompatibility as an important essential property of intravenous carriers has also been used to examine the safety of HBOCs. According to the literature the threshold value of hemolysis rate of HBOCs is below 5%, which indicates that the microcapsules do not interact with other components of the blood and therefore do not have adverse effects on the blood after introduction [40,44,51,59]. The Hb-NPs and Hb/PDA-NPs did not hemolysis to occur, even at higher concentrations [40,44]. The hemolysis rates of PLGA/Hb^PDA^/(CeO_2_-NPs)-NCs and PLGA/Hb^PDA^/(CeO_2_-NPs)-NCs were both well below 5% under the wrapping of cell membranes [51,55].

## 6 Conclusion

HOBCs have an important role as a blood substitute used clinically to maintain normal physiological functions of the body. With the continuous development and optimization of synthesis technology, it is now common to combine one or more of the above methods to synthesize the required HBOCs with the advantages of higher oxygen-carrying capacity, higher stability, improved half-life, and lower toxicity. However, there are some unavoidable drawbacks (Table 2). For example, although the coprecipitation method for the synthesis of HBOCs is simple in operation, without the introduction of toxic substances and with greater solubility in various buffers, its stability is poor compared to several other methods, so the cross-linking agent and the formation of microcapsules are introduced on this basis. However, the introduction of cross-linking agents largely causes vasoconstriction and has a potential risk of causing hypertension. In addition, the formation of hemoglobin into polymers leaves relatively small molecules or unreacted Hb, and when liposomes are encapsulated, the formation of membranes requires the addition of other substances to fill the shell.

**Table 2:** Merits and disadvantages of different synthesis methods.

Method of synthesis	Merits	Disadvantages
coprecipitation	simple, no toxic substances introduced, high encapsulation efficiency and solubility in a polar solvent	lower stability
cross-linking	increased half-life, improved stability, and oxygen-carrying capacity, reduced protein immunogenicity	blood pressure rises, vasoconstriction
polymer	increased half-life, smaller side effects, and larger particle size	residual unreacted Hb
liposome	uniform particle size, good stability	cumbersome steps

HBOCs were initially developed and used as blood substitutes as an ideal class of oxygen carriers for early applications in hemorrhagic shock. However, the ethical problems associated with the use of modified HBOCs in emergency care, the significant cardiovascular dysfunction and the apparent increased mortality caused by them led to the extended use of most HBOCs in organ preservation fluids. In organ storage, HBOCs have positive effects such as anti-apoptotic, anti-inflammatory and anti-proliferative. Not only that, HBOCs improve oxygen supply and preserve optimal metabolic activity, reduce oxidation-mediated tissue damage and enhance liver capacity storage, but also have positive effects in preserving and improving marginal organs. Mechanistic studies related to vascular resistance, methemoglobin and oxidative damage in transplants and recipients should also be closely investigated in future studies. In the future, according to the characteristics of storing different types of organs, we will screen the synthesis method that is most suitable for storing HBOCs of that type of organ, so as to construct the optimized storage solution.

## Author contribution statement

All authors listed have significantly contributed to the development and the writing of this article.

1) conceived and designed the experiments.

2) performed the experiments.

3) analyzed and interpreted the data.

4) contributed reagents, materials, analysis tools or data.

5) wrote the paper.

## Data availability statement

Data will be made available on request.

## Additional information

No additional information is available for this paper.

## Declaration of competing interest

The authors declare that they have no known competing financial interests or personal relationships that could have appeared to influence the work reported in this paper.
